# Overcoming the Trade‐Off Between Magnetic Coupling and Electrical Insulation in Soft Magnetic Materials via Nanochain Engineering

**DOI:** 10.1002/advs.202517270

**Published:** 2025-12-16

**Authors:** Dingrong Zuo, Lichen Liu, Haibo Ke, Rongsheng Bai, Huaping Ding, Jing Zhou, Zhenxiang Cheng, Peng Yu, Weihua Wang

**Affiliations:** ^1^ College of Physics and Electronic Engineering Chongqing Normal University Chongqing 401331 China; ^2^ Songshan Lake Materials Laboratory Dongguan 523808 China; ^3^ Institute for Superconducting and Electronic Materials Faculty of Engineering and Information Sciences Innovation Campus University of Wollongong Squires Way NSW 2500 Australia; ^4^ Institute of Physics Chinese Academy of Sciences Beijing 100190 China

**Keywords:** electrical insulation, high‐frequency soft magnetic materials, magnetic coupling, nanochain engineering, nanoparticles, soft magnetic composites

## Abstract

The development of high‐frequency soft magnetic materials (HFSMMs) that simultaneously achieve high saturation magnetization strength (*M*_s_) and high electrical resistivity (*ρ*) represents a significant challenge, primarily due to the inherent trade‐off between magnetic coupling and electrical insulation. Herein, a novel approach is presented to address this challenge through the self‐isolated Fe/FeCo nanochains with continuous oxide encapsulation, fabricated via a magnetic field‐assisted chemical synthesis combined with in situ oxidation. The unique nanochain architecture facilitates robust magnetic coupling between nanoparticles, while an ultrathin oxide layer (≈10 nm) provides effective electrical isolation, enhancing resistivity. Detailed structural and theoretical analyses demonstrate that the amorphous‐crystalline interfaces promote spin polarization through charge recombination, thereby boosting magnetization, while the metal‐oxide interfaces confine eddy currents within individual nanoparticles, significantly reducing high‐frequency core losses. The resulting nanochain soft magnetic composites (nanochain‐SMCs) exhibit an exceptional property: Fe nanochains achieve *M*_s_ = 149.2 emu g^−1^ and *ρ* = 0.86 Ω·m, while FeCo nanochains reach *M*_s_ = 172.1 emu g^−1^ and *ρ* = 0.41 Ω·m, alongside outstanding frequency stability. This study highlights the synergy between magnetic and charge dynamics in low‐dimensional materials, providing a new insight for the development of HFSMMs.

## Graphical abstract

This study employs a nanochain engineering approach combined with an in situ oxidation strategy to fabricate self‐insulating iron‐based magnetic nanochains, addressing the challenge of balancing magnetic coupling and electrical insulation in soft magnetic materials. The ultra‐thin native oxide layer (≈10 nm) effectively confines eddy currents while enhancing electrical resistivity, and simultaneously maintains strong magnetic coupling between nanoparticles.

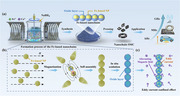

## 1 Introduction

The burgeoning demand for high‐frequency and integrated electronic devices has firmly established high‐frequency soft magnetic materials (HFSMMs) as indispensable components for power applications, including high‐frequency transformers and inductors.^[^ ^1^ ^]^ To perform effectively, HFSMMs must exhibit high‐frequency stable permeability, high saturation magnetization strength (*M*_s_), and high resistivity. Conventional soft magnetic composites (SMCs) typically rely on micron‐sized magnetic particles coated with high‐resistivity materials like SiO_2_ or resin to provide electrical insulation, thereby mitigating eddy current losses under the high‐frequency alternating magnetic field. However, the insulation layers formed by this method are typically thick (> 100 nm), while increasing the coating agent content can enhance insulation, these thick layers hinder interparticle magnetic exchange coupling, significantly compromising *M*_s_.^[^ ^2^, ^3^ ^]^ In contrast, soft magnetic ferrites boast high resistivity (up to 10^12^ µΩ·cm) and are effective in the gigahertz range, yet their intrinsically low *M*_s_ (< 50 emu g^−1^) limits their use in miniaturized inductors. Achieving both high *M*_s_ and high resistivity in soft magnetic materials fundamentally requires balancing the mutually restrictive properties of magnetic coupling and electrical insulation.^[^ ^4^, ^5^ ^]^ At the microscopic level, the high *M*_s_ relies on strong magnetic coupling, such as direct exchange interaction between 3*d* electrons in Fe/Co‐based materials, which necessitates a high degree of alignment of atomic magnetic moments.^[^ ^6^, ^7^ ^]^ However, such strongly coupled systems often exhibit low resistivity and large magnetic crystal anisotropy, leading to significant eddy current and hysteresis losses at high frequencies.^[^ ^8^ ^]^ While electrical insulation, such as superexchange interactions in ferrites, can suppress eddy currents through high resistivity, it inherently compromises *M*_s_ or permeability by localizing electronic states and weakening long‐range magnetic order. This inherent conflict stems from the fact that magnetic coupling promotes collective magnetic moments response, while electrical insulation suppresses charge carrier migration, resulting in an inverse trade‐off in conventional materials.^[^ ^9^ ^]^ Therefore, circumventing this trade‐off requires innovative architectures enabling spatial decoupling of magnetic exchange and electrical conduction pathways.

Recent advances in nanoscale magnetic materials offer a potential solution.^[^ ^10^ ^]^ The chemical approach enables the low‐cost batch preparation of magnetic nanoparticles. For instance, chemically reduced Fe‐based magnetic nanoparticles exploit size effects to achieve high *M*_s_ and cut‐off frequency exceeding 1 GHz, while their reduced size diminishes eddy current loss at high frequencies.^[^ ^11^, ^12^ ^]^ However, such chemically synthesized nanoparticles often suffer from uncontrolled aggregation due to van der Waals interactions. Additionally, unavoidable natural oxidation in air, caused by irregular clustering, leads to uneven oxidation, seriously weakening the magnetic properties. Preventing disordered clustering and enhancing resistivity necessitates the use of dispersant additives during the preparation of magnetic nanoparticles and insulation coatings prior to SMC compaction.^[^ ^13^, ^14^ ^]^ This inevitably introduces excessive non‐magnetic components, degrading magnetic properties and limiting practical implementation. Consequently, conventional approaches using magnetic nanoparticles fail to resolve the fundamental *M*_s_‐resistivity trade‐off. Theoretically, achieving both high *M*_s_ and high resistivity requires an architecture where magnetic nanoparticles are ordered with minimal spacing to enhance collective magnetic response while being isolated by a high‐resistivity material that ensures both strong magnetic coupling and electrical insulation.^[^ ^7^ ^]^ This underscores the need for strategies that achieve both nanoparticle ordering and intrinsic insulation without sacrificing magnetic functionality.

Herein, we report a strategy to overcome this limitation by using “self‐isolated magnetic nanochains”. By integrating magnetic field‐assisted assembly with intrinsic oxidation, chemically reduced Fe/FeCo nanoparticles self‐assemble into chain‐like structures, while a spontaneously formed high‐resistivity oxide layer provides electrical isolation without the need for conventional thick coatings. Crucially, this self‐isolated architecture confines major eddy currents to intra‐particle pathways under an alternating magnetic field, effectively suppressing eddy current loss while preserving strong interparticle magnetic coupling along the chain axis. Consequently, the resultant nanochain‐SMCs exhibit simultaneous high *M*_s_, high resistivity, and remarkably high‐frequency stability, with a cut‐off frequency exceeding 1 GHz. The Fe‐based nanochain materials prepared by this research can be utilized for developing high‐frequency soft magnetic electronic devices due to their performance advantages and stability.

## 2 Results and Discussion

### 2.1 The Preparation and Structural Characterization of Nanochain‐SMCs

**Figure 1:**
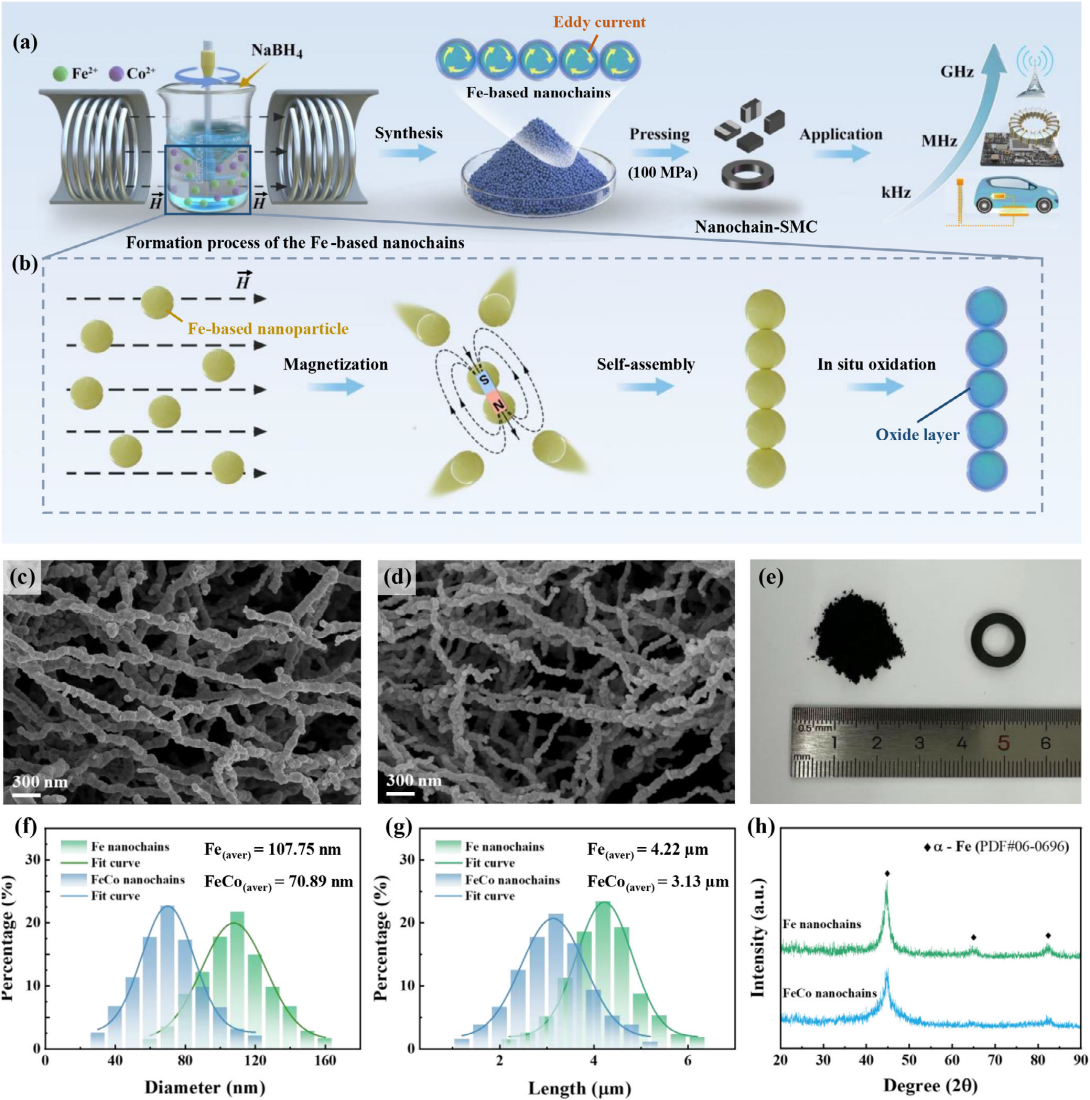
Synthesis and structural Characterization of the Fe‐based magnetic nanochains. a) Schematic overview of the magnetic‐field‐assisted synthesis process, illustrating the nanochain architecture with confined eddy current effects, subsequent composite formation (nanochain‐SMC), and potential device applications. b) Proposed formation mechanism of field‐aligned nanochains through magnetic dipole assembly under a uniform magnetic field ($\vec{H}$). c, d) SEM images demonstrating the chain‐like morphology of the Fe (c) and FeCo (d) nanochains. e) Macroscopic view of as‐synthesized Fe nanochain powders and corresponding toroidal SMC. f, g) Statistical size distributions showing diameter (f) and length (g) of the nanochains determined by Gaussian fitting. h) XRD patterns confirming the coexistence of amorphous and α‐Fe crystalline phases in the nanochains.

The Fe and FeCo nanochains are synthesized through a magnetic field‐assisted chemical reduction process coupled with in situ oxidation. **Figure** **1**a presents a schematic overview that simultaneously captures the nanochain synthesis process, nanostructure design principles, the eddy current confinement mechanism in nanochain‐SMCs, and their potential multifunctional applications. The formation mechanism (Figure 1b) reveals that chemically reduced Fe/FeCo nanoparticles serve as magnetic building blocks that spontaneously organize into chain‐like structures under magnetic guidance. When exposed to an external magnetic field, the Fe‐based nanoparticles become magnetized, generating magnetic dipoles that interact through mutual dipole‐dipole attraction.^[^ ^15^ ^]^ This interaction creates a reinforced dipole moment that drives sequential assembly, with additional nanoparticles aligning along the dipole axis to propagate nanochain growth. Following assembly, the nanochains undergo in situ oxidation to form a nanoscale oxide layer that effectively isolates individual nanoparticles while enhancing the material's overall resistivity. Microstructural characterization shows distinct morphological features for both Fe (Figure 1c) and FeCo (Figure 1d) nanochains, with corresponding elemental mapping presented in Figure S1 (Supporting Information). Gaussian fitting analysis of dimensional parameters (Figure 1f,g) reveals that Fe nanochains exhibit an average diameter (*D*) of 107.75 nm and length (*L*) of 4.19 µm (aspect ratio *L*/*D* ≈ 39), while the FeCo nanochains demonstrate smaller dimensions (*D* = 70.89 nm, *L* = 3.15 µm) but a higher aspect ratio (*L*/*D* ≈ 44). This increased anisotropy in the FeCo system arises from stronger magnetic exchange coupling, where reduced nanoparticle size maintains adequate magnetic dipole moments for chain assembly through dipole‐dipole interactions.^[^ ^16^ ^]^ The self‐assembly of magnetic nanochains is triggered by the magnetization of nanoparticles under an external field. An excessively high field strength does not markedly change the resulting nanochain morphology but may slightly enhance the aspect ratio. In contrast, a field that is too low leads to inadequate nanoparticle magnetization, promoting disorder and yielding nanochains with a reduced aspect ratio. This work confirms that a 10 mT external field is sufficient to produce highly ordered Fe‐based nanochains. These nanochains can be directly compacted into bulk composites (nanochain‐SMCs) under moderate pressure (100 MPa), significantly simplifying the manufacturing process. Figure 1e shows the photo of both Fe nanochain powders and the resulting toroidal Fe nanochain‐SMC. X‐Ray Diffraction (XRD) analysis (Figure 1h) reveals predominantly amorphous matrices with minor α‐Fe crystalline phases in both systems, indicating spontaneous room‐temperature crystallization during synthesis.

**Figure 2:**
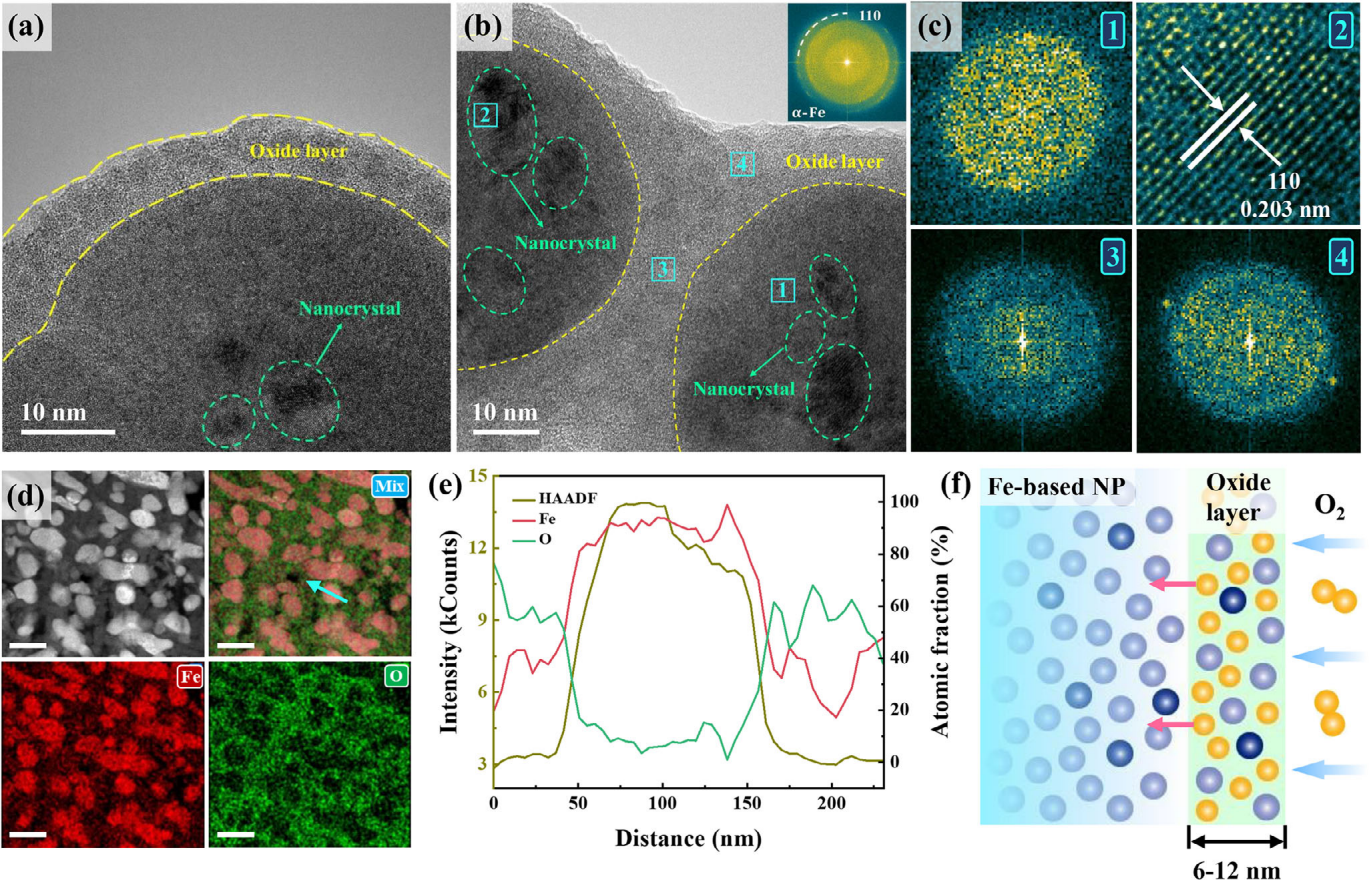
Microstructure characterization of the Fe nanochains. a, b) TEM images revealing the nano‐oxide layer and the amorphous‐crystalline composite structure of the Fe nanochains. c) FFT (numbers 1, 3, and 4) and HR‐TEM (number 2) images for the different regions marked as the blue square in (b), providing crystallographic information at the nanoscale. d) HAADF images and elemental maps (scalebar represents 200 nm) of the Fe nanochain‐SMC, illustrating the spatial distribution of constituent elements. e) EDS line scan profile across an individual Fe‐based nanoparticle (indicated by the blue arrow in (d)), quantifying its elemental composition. f) Schematic representation of the oxide layer formation mechanism on the surface of an Fe‐based nanoparticle (NP).

**Figure 3:**
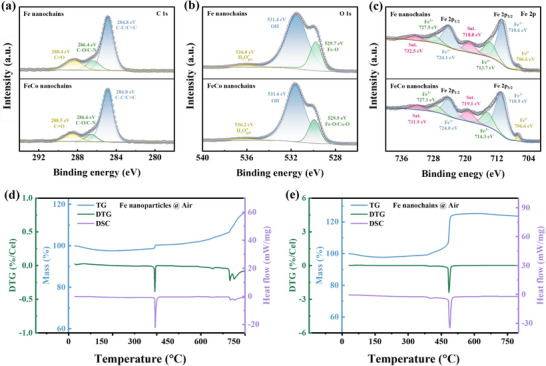
Surface chemical states and thermal stability of the nanochains. a–c) XPS spectrum of the nanochains, including the C 1s (a), O 1s (b), and Fe 2p (c) core‐level regions, which elucidate the surface chemical states and bonding environments. d, e) Simultaneous TG‐DSC thermal analysis of the Fe nanoparticles (d) and the Fe nanochains (e), the heating rate is 10 K min^−1^.

Transmission Electron Microscopy (TEM) examination of Focused Ion Beam (FIB)‐prepared samples provides detailed microstructural insights into both Fe nanochains and Fe nanochain‐SMCs. **Figure** **2**a clearly shows Fe‐based nanoparticles (dark contrast) encapsulated by a ≈ 10 nm amorphous oxide layer (light contrast), with visible intracrystalline nanocrystalline phases. The volume fraction of the nanocrystalline phase embedded in the amorphous matrix is estimated to be in the range of 10‐20%. The chain architecture (Figure 2b) demonstrates that nanochains consist of magnetically coupled nanoparticles separated by oxide layers, maintaining ≈10 nm interparticle spacing rather than direct contact. The inset Fast Fourier Transform (FFT) image in Figure 2b displays a distinct diffraction ring corresponding to the α‐Fe (110) plane. Further analysis of marked regions in Figure 2b (Figure 2c) reveals that intra‐particle (Region 1) and extra‐particle oxide (Region 3) areas exhibit amorphous characteristics through diffuse FFT halos, while Region 2 shows crystalline lattice fringes with 0.203 nm spacing matching α‐Fe (110) planes ‐ consistent with XRD results.^[^ ^17^, ^18^ ^]^ Region 4 exhibits faint lattice patterns in the FFT image, indicating the presence of localized nanocrystallites within the predominantly amorphous oxide layer. Similar microstructural features are observed for FeCo nanochains and FeCo nanochain‐SMC (Figure S2, Supporting Information). High‐Angle Annular Dark Field (HAADF) imaging of Fe nanochain‐SMC (Figure 2d) confirms the spatial distribution of magnetic nanoparticles (light‐colored region) isolated by the oxide layer (dark‐colored region), through compaction‐induced deformation caused some deviation from ideal chain alignment. Linear scanning across nanoparticles (Figure 2e, following blue arrows in Figure 2d) reveals distinct oxygen gradients, with < 5% oxygen content within nanoparticles compared to ≈60% in the oxide layer. X‐ray Photoelectron Spectroscopy (XPS) analysis (**Figure** **3**c) identifies amorphous iron oxide (Fe_3_O_4_ and α‐Fe_2_O_3_) as the predominant oxide layer component.

At room temperature, Fe‐based nanoparticles exposed to air typically develop a self‐limiting oxide shell. This shell–core structure consists of a metallic Fe° core encapsulated by a mixed‐valence oxide layer (commonly composed of Fe_3_O_4_ and α‐Fe_2_O_3_) with a thin outermost layer of hydrated or hydroxylated oxides (FeOOH) that may exist in reversible or irreversible forms.^[^ ^14^ ^]^ Such a shell‐core configuration has been consistently verified by TEM, XPS, and Mössbauer spectroscopy, and is further supported by kinetic models as well as molecular‐ and atomistic‐scale simulations.^[^ ^19^, ^20^ ^]^ Collectively, these findings establish a coherent mechanistic framework in which the early‐stage oxidation is dominated by surface electric‐field‐driven ion migration, while at longer timescales, diffusion and interfacial thermodynamic equilibrium govern the final chemical state of the shell. The classical Cabrera‐Mott model effectively explains the rapid initial growth followed by self‐limiting behavior observed in ultrathin (few‐nanometer) oxide layers (Figure 2f).^[^ ^21^, ^22^ ^]^ Meanwhile, the microscopic processes of oxygen penetration and metal–oxygen interdiffusion determine the ultimate phase composition and oxide thickness, both of which are strongly influenced by the coupled effects of particle size, temperature, and humidity. In the present work, Fe/FeCo nanochains that have reached thermodynamic stability under normal temperature and pressure conditions exhibit an oxide shell thickness of ≈6–12 nm (Figure 2a,b). Although this thickness cannot be precisely controlled, it remains significantly thinner than the insulating coatings typically used in conventional magnetic powder cores. As a result, the native oxide layer not only provides effective electrical isolation between adjacent nanoparticles but also enhances their interparticle magnetic coupling.

### 2.2 Native Oxidation State and Thermal Stability Characterization

XPS analysis provides detailed surface chemistry information for both Fe and FeCo nanochains. High‐resolution C 1s spectra (Figure 3a) deconvolve into three components for both systems: C─C/C═C (248.8 eV), C─O/C─N (≈286 eV), and C═O (≈288 eV).^[^ ^23^ ^]^ The O 1s spectra (Figure 3b) display characteristic peaks for metal oxide O^2−^ (≈529 eV), surface hydroxyl groups (≈531.4 eV), and physisorbed H_2_O (≈536.0 eV).^[^ ^24^ ^]^ Fe 2p spectra (Figure 3c) confirm predominantly oxidized surface states (Fe^2+^: ≈710/724 eV, Fe^3+^: ≈714/727 eV) with minor metallic Fe^0^ (≈706 eV), alongside characteristic shake‐up satellite peaks (≈719/732 eV).^[^ ^25^, ^26^, ^27^ ^]^ These results collectively establish iron oxide (Fe_3_O_4_ and α‐Fe_2_O_3_) as the primary oxide layer constituent, formed through surface oxidation of Fe nanoparticles in both solution and air environments. For the FeCo nanochains, metallic Co underwent a similar oxidation reaction, resulting in an oxide layer containing minor amounts of the amorphous mixture of CoO and Co_3_O_4_, as evidenced by Co 2p XPS spectra in Figure S3 (Supporting Information).

The thermal stability of Fe nanochains was evaluated using simultaneous Thermogravimetric Analysis and Differential Scanning Calorimetry (TG‐DSC) and compared with that of disordered Fe nanoparticles. Figure S4 (Supporting Information) shows the disordered Fe (Figure S4a, Supporting Information) and FeCo (Figure S4b, Supporting Information) nanoparticles synthesized without an applied magnetic field. When heated from room temperature to 800 °C under Ar, both samples reached their minimum mass at ≈394 °C, with mass retentions of 96.72% for the Fe nanoparticles (Figure S5a, Supporting Information) and 96.92% for the Fe nanochains (Figure S5b, Supporting Information). This mass reduction is mainly due to the decomposition and evolution of oxygen from the oxides. Furthermore, the Fe nanoparticles and the Fe nanochains were heated from room temperature to 800 °C in air. As shown in Figure 3d, the Fe nanoparticles undergo a dramatic oxidation event ≈391 °C, leading to a rapid mass increase. The mass subsequently continues to rise gradually with temperature, reaching 116%. In contrast, this oxidative behavior for the Fe nanochains occurs at a higher temperature of ≈485 °C, where their mass increases rapidly to ≈125% and then stabilizes, showing no significant change with further heating (Figure 3e). This discrepancy arises from the structural inhomogeneity of the disordered Fe nanoparticles (Figure S4a, Supporting Information), whereas the structurally uniform and coherent architecture of the Fe nanochains provides enhanced thermal stability and a more consistent oxidation behavior. Furthermore, the Fe nanochains show no notable mass increase within 0–400 °C, indicating that the thickness of their oxide layer did not grow further (Figure 3e). The magnetization curves of Fe/FeCo nanochains at different temperatures indicate that upon a 100 °C increase from room temperature (300 to 400 K), the magnetization of the Fe and FeCo nanochains decreased by 6.8% and 10.4%, respectively (Figure S6, Supporting Information). The Fe/FeCo nanochains do not exhibit a single, well‐defined Curie temperature but rather demonstrate a multiphase and continuous magnetic order transition. This behavior is attributed to the compositional and structural complexity of the nanochains. In summary, Fe‐based nanochain materials demonstrate considerable thermal stability, retaining moderate magnetic properties over the 0–100 °C range, thereby establishing a foundation for their use in high‐frequency soft magnetic devices.

### 2.3 Comprehensive Soft Magnetic Properties

**Figure 4:**
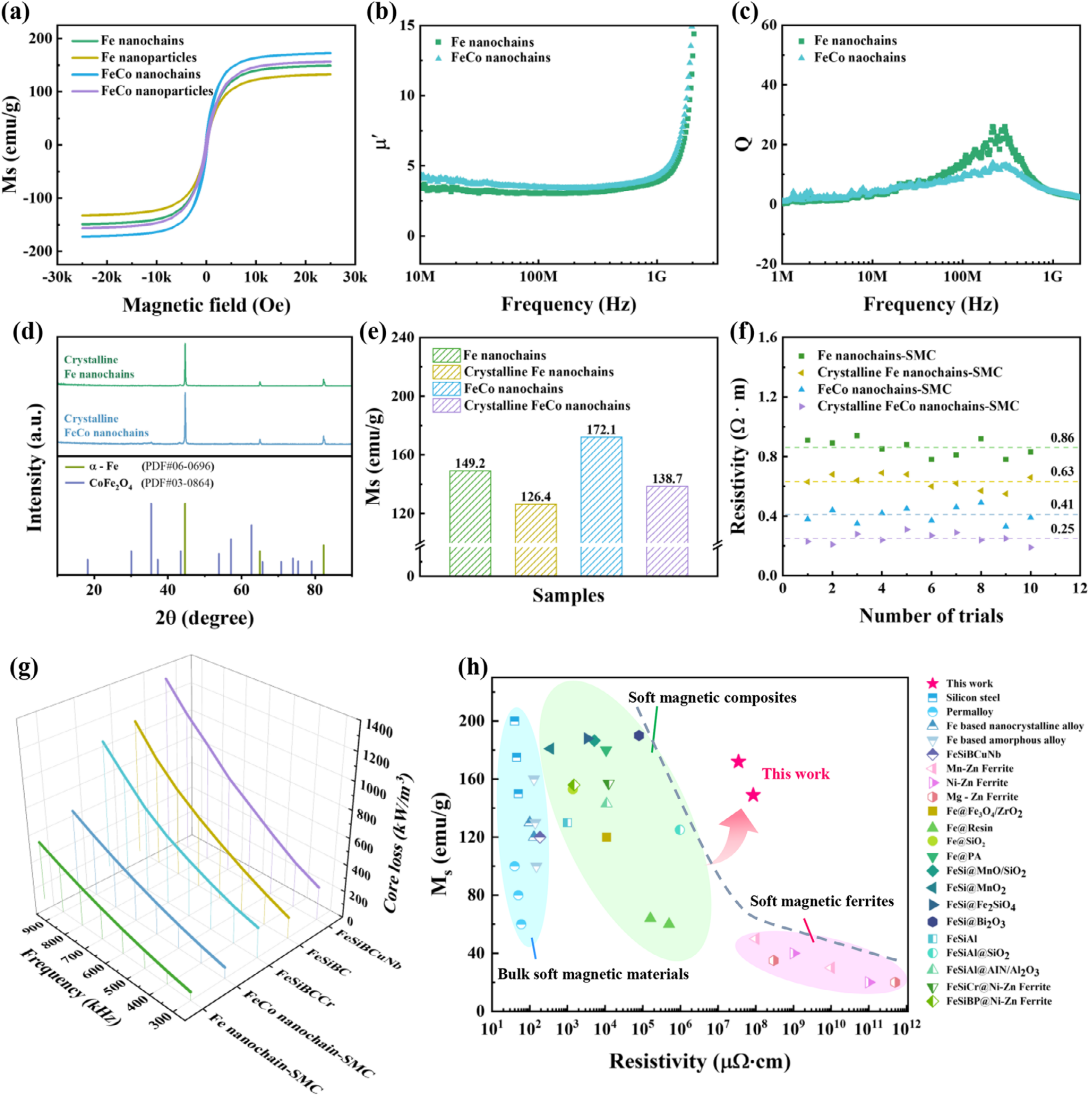
Magnetic and electrical properties of the nanochains and nanochain‐SMCs. a) Magnetic hysteresis loops comparing the magnetization behavior of the Fe‐based nanochains and nanoparticles (Fe‐based nanoparticles are obtained by chemical reduction without the external magnetic field). b, c) Frequency dependence of real permeability (*µ*ʹ) and quality factor (Q) of the nanochains. d) XRD patterns of the crystalline nanochains (obtained by annealing the nanochains at 500 °C for 0.5 h). e) Saturation magnetization strength (*M*_s_) comparing the nanochains and crystalline nanochains. f) Resistivity comparing the nanochain‐SMCs and the crystalline nanochain‐SMCs. g) High‐frequency core loss comparison of the nanochain‐SMCs and commercial SMCs at 20 mT. h) Saturation magnetization (*M*_s_) versus resistivity.

Our systematic investigations of the magnetic and electrical insulation properties of the Fe‐based nanochains reveal significant enhancement in their characteristics. Magnetic hysteresis loops analysis (**Figure** **4**a) demonstrates substantial improvement in *M*_s_ for the field‐assisted nanochain compared to their randomly aggregated nanoparticle counterparts synthesized without magnetic guidance. Specifically, Fe nanochains exhibit *M*_s_ = 149.2 emu g^−1^ compared to 132.8 emu g^−1^ for nanoparticles, while the FeCo nanochains achieve 172.1 versus 153.6 emu g^−1^ for their nanoparticle counterparts. This ≈12–15% enhancement arises from reduced interparticle distances that strengthen magnetic exchange coupling within the chain configurations. Frequency‐dependent permeability (*µ*ʹ) and the quality factor (Q) measurements (Figure 4b,c) clearly show that both systems exhibit cut‐off frequencies exceeding 1 GHz, consistent with previous studies on Fe‐based nanochains.^[^ ^28^ ^]^ At 1 GHz, *µ*ʹ reaches 4.18 for Fe and 3.54 for FeCo, with the latter's higher *M*_s_ attributed to stronger Fe─Co exchange interactions compared to Fe‐Fe pairs.^[^ ^29^ ^]^ The optimal operating frequency for the Fe‐based nanochain‐SMCs is identified in the 100–500 MHz range, indicating suitability for RF power applications such as inductor devices. Furthermore, the coercivity at different frequencies measurements indicate that the Fe/FeCo nanochain‐SMCs possess a notably low coercivity, which approaches a steady state within the frequency range of 300–1000 kHz (Figure S7, Supporting Information). Such behavior suggests that this material is capable of maintaining stable B‐H characteristics under high‐frequency (> MHz) operating conditions, making it highly suitable for power electronic devices.

The room‐temperature spontaneous crystallization observed in the Fe‐based nanochains results in the formation of nanocrystals within the amorphous nanoparticles, as evidenced in Figure 2a,b. This unique amorphous‐crystalline composite structure demonstrates superior magnetic properties and resistivity compared to fully crystalline counterparts. Comparative analysis between as‐synthesized nanochains and vacuum‐annealed (500 °C for 0.5 h) crystalline controls reveals that the crystalline nanochains (Figure 4d) exhibit the α‐Fe structure with minor metal oxide phase (Fe_3_O_4_ and CoFe_2_O_4_). Magnetic measurements (Figure 4e) reveal significantly enhanced *M*_s_ in amorphous‐crystalline composite structures: 149.2 emu g^−1^ (Fe) and 172.1 emu g^−1^ (FeCo) versus 126.4 and 138.7 emu g^−1^ in crystalline analogues, representing 15‐18% improvements. Correspondingly, electrical resistivity (Figure 4f) increases from 0.63 to 0.86 Ω·m and from 0.25 to 0.41 Ω·m in Fe and FeCo composite structures, respectively. This dual enhancement originates from the disordered nature of the amorphous structure, impeding electron mobility while preserving exchange‐coupled nanocrystalline regions.

Core loss curve (Figure 4g) demonstrates that Fe‐based nanochain‐SMCs exhibit significantly lower loss than several commercial SMCs in the frequency range of 300–1000 kHz at *B*_m_ = 20 mT. The near‐linear frequency dependence of core loss for both the Fe and FeCo nanochain‐SMCs suggests frequency‐insensitive loss characteristics optimal for high‐frequency applications. Analysis of the loss composition in the Fe/FeCo nanochain‐SMCs across different frequencies reveals that the proportion of eddy current loss gradually increases with rising frequency, eventually becoming the dominant contributor (Figure S8, Tables S1 and S2, Supporting Information). This highlights that reducing eddy current losses is crucial for high‐frequency soft magnetic materials.^[^ ^30^ ^]^ Since the SMCs are pressed from a large amount of magnetic particle powder, the total eddy current loss (*P*_e_) consists of inter‐particle ($P_{e}^{\mathrm{inter}}$) and intra‐particle ($P_{e}^{\mathrm{intra}}$) contributions governed by the following equation:^[^ ^31^, ^32^ ^]^

(1) $$P_{e}=P_{e}^{\mathrm{inter}}+P_{e}^{\mathrm{intra}}=\frac{\pi^{2}d_{e}^{\;2}B_{m}^{2}}{\beta \rho_{\mathrm{SMC}}}f^{2}+\frac{\pi^{2}d^{2}B_{m}^{2}}{20\rho }f^{2}$$

where *B*_m_ is the magnitude of magnetic induction, *f* is the frequency of the alternating magnetic field, *ρ* is the resistivity of the particle, *ρ*_SMC_ is the resistivity of the SMC, and *d*_e_ respectively represent the magnetic particle size and the effective diameter of eddy current, which can be regarded as the thickness of the SMC, *β* is the geometric coefficient. The exceptional loss reduction in nanochain‐SMCs at high frequencies arises from dual structural advantages: high‐resistivity oxide layers, elevating overall *ρ*_SMC_ and nanoscale particle dimension (*d*), minimizing intraparticle eddy paths.

Comprehensive performance benchmarking of the Fe‐based nanochain‐SMCs against conventional soft magnetic materials is presented in Figure 4h; the specific *M*_s_ and *ρ* data for the soft magnetic composite materials can be found in Table S3 (Supporting Information). It reveals an intrinsic materials paradox that existing soft magnetic materials systems cannot simultaneously achieve high *M*_s_ and elevated resistivity for high‐frequency operation. For instance, the silicon steel attains *M*_s_ > 200 emu g^−1^ but suffers from low resistivity (100 µΩ·cm), limiting applicability to sub‐400 Hz regimes. Conversely, soft magnetic ferrites offer ultrahigh resistivity (up to 10^12^ µΩ·cm), enabling GHz operation, yet their low *M*_s_ (< 50 emu g^−1^) and modest Curie temperature (≈200 °C) impede miniaturized inductor integration.^[^ ^33^ ^]^ Conventional SMCs enhance resistivity via particle coating, but increased insulation thickness catastrophically attenuates magnetic coupling and *M*_s_. Remarkably, our nanochain‐SMCs overcome this fundamental trade‐off: Fe systems achieve the *M*_s_ = 149.2 emu g^−1^ and the resistivity is 0.86 Ω·m, while FeCo counterparts reach 172.1 emu g^−1^ and 0.41 Ω·m. This exceptional combination of performance characteristics surpasses the majority of existing soft magnetic materials, demonstrating considerable potential for high‐frequency applications.

### 2.4 The Physical Mechanism of the Excellent Performance

**Figure 5:**
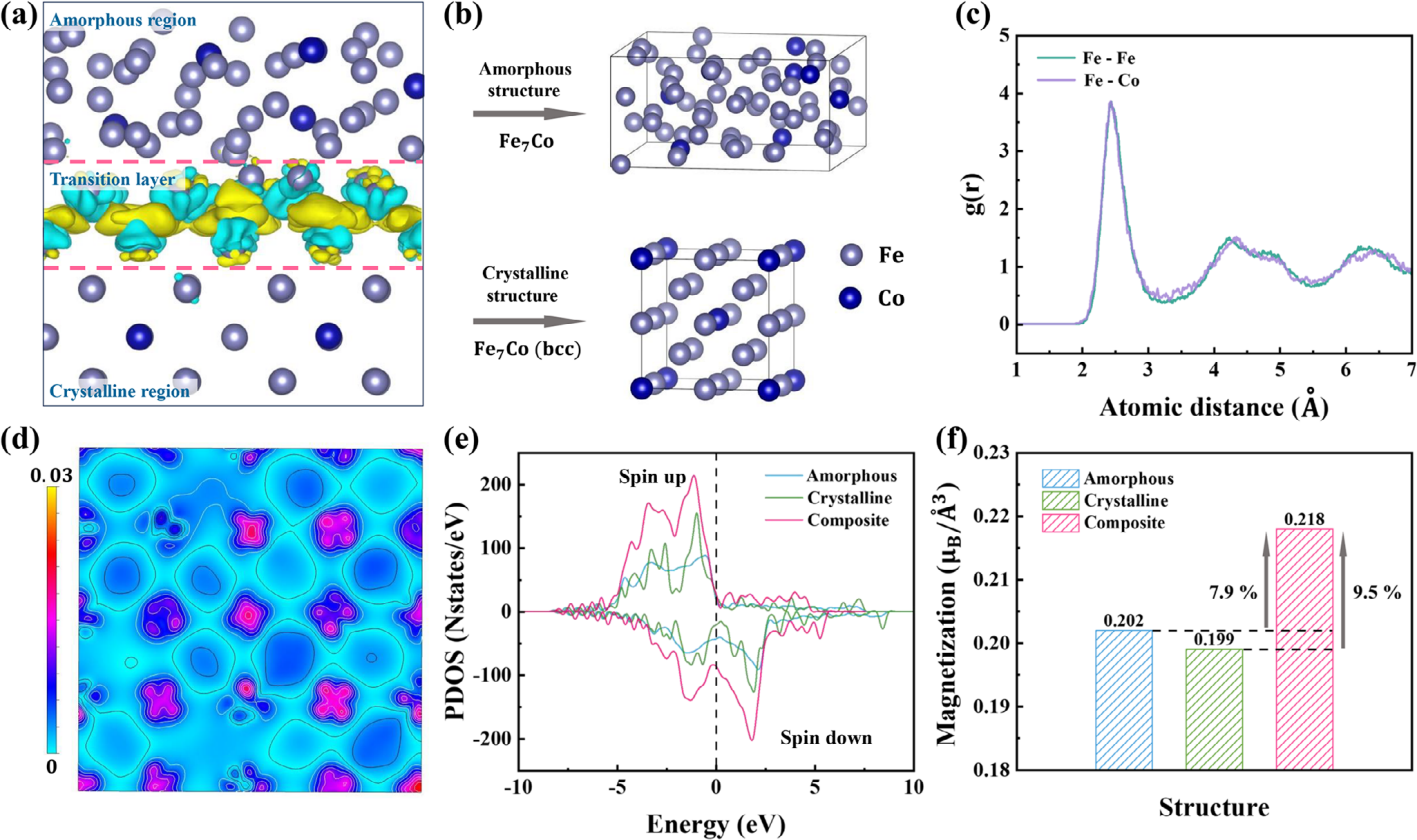
First‐principle investigation of the amorphous‐crystalline composite structure. a) 3D charge density difference of the transition layer between the amorphous region and the crystalline region. b) Structure of the amorphous region (the Fe_7_Co amorphous structure obtained by rapid annealing of the Fe_7_Co crystal structure) and crystalline region in (a). c) Pair distribution functions (PDF) of the Fe_7_Co amorphous structure. d) 2D charge density difference of the transition layer. e, f) PDOS and Magnetization comparing the amorphous, crystalline, and overall composite structures in (a).

First‐principle calculations were performed to elucidate the enhancement mechanism in amorphous‐crystalline composite structures. The 3D charge density differences (**Figure** **5**a) reveal significant charge redistribution (yellow regions: accumulation; cyan regions: depletion) at the amorphous‐crystalline interface. As shown in Figure 5b, we employ compositionally identical Fe_7_Co amorphous and crystalline (110) structural models to construct the heterostructure. The pair distribution function (PDF) of the above amorphous region (Figure 5c) confirms successful modeling of long‐range disorder.^[^ ^16^ ^]^ The 2D charge density difference mapping at the interface (Figure 5d) shows decreased charge density within white contours versus increased charge density in black contours. The Partial Density of States (PDOS) analysis (Figure 5e) demonstrates spin asymmetry, while the DOS at the Fermi energy level is dominated by spin‐down Fe 3d unpaired electrons, suggesting interfacial charge recombination enhances spin polarization.^[^ ^34^, ^35^, ^36^ ^]^ Crucially, magnetization calculations per unit volume (Figure 5f) show the composite structure exhibits 7.9% and 9.5% higher magnetic moments than pure amorphous and crystalline domains, respectively. This aligns with experimental observations where control of α‐Fe phase nanocrystal precipitation optimizes the magnetic properties.^[^ ^37^, ^38^, ^39^ ^]^

**Figure 6:**
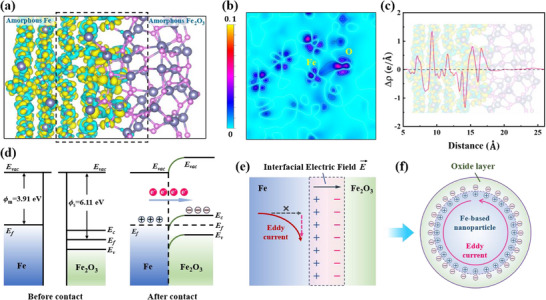
First‐principle study of the metal‐oxide interface. a) 3D charge density difference of the interface between the amorphous Fe and the amorphous Fe_2_O_3_. b) 2D charge density difference of the interface (marked as the dashed rectangle in (a)). c) Average charge density difference along the direction perpendicular to the interface. d) Schematic diagram of the band structure of the amorphous Fe and the amorphous Fe_2_O_3_. e) Confining effect of the metal‐oxide (Fe‐Fe_2_O_3_) interfacial electric field on eddy currents. f) Schematic diagram of the vortex path of the Fe‐based nanoparticle with an oxide layer.

Furthermore, the confinement mechanism of eddy currents at the metal‐oxide interface was investigated via first‐principles calculations (**Figure** **6**). An amorphous model of the Fe‐Fe_2_O_3_ interface was constructed (Figure S9, Supporting Information). Figure 6a presents the 3D charge density differences (yellow regions: accumulation; cyan regions: depletion) of the Fe‐Fe_2_O_3_ interface, with the isosurface value set at 0.01 eV. Figure 6b displays the 2D charge density differences near the interface between amorphous Fe and amorphous Fe_2_O_3_ within the region denoted by the dashed black rectangle in Figure 6a. Here, black contours represent regions of electron accumulation, while white contours indicate regions of electron depletion. The scale bar corresponds to a range of 0–0.1 eV. The analysis reveals a tendency for electrons from Fe atoms in the metallic region to migrate toward oxygen atoms in the Fe_2_O_3_ region. Figure 6c demonstrates the charge redistribution across the interface between amorphous metallic Fe and amorphous oxide Fe_2_O_3_. The results reveal that along the direction perpendicular to the interface, ≈2–3 atomic layers within the metallic region exhibit a tendency for electron depletion, while ≈2 atomic layers in the oxide region show a tendency for electron accumulation. The charge transfer at the metal‐oxide interface can be attributed to the work function difference between the materials. Figure 6d presents the band structure of amorphous Fe and amorphous Fe_2_O_3_; the corresponding partial density of states (PDOS) information can be found in the (Figure S10, Supporting Information). In Figure 6d, *E*_vac_ represents the vacuum energy level, *E*_c_ denotes the conduction band minimum (CBM), *E*_f_ signifies the Fermi level, and *E*_v_ indicates the valence band maximum (VBM). Since the work function of metallic Fe (*ϕ*_m_ = 3.91 eV) is lower than that of Fe_2_O_3_ (*ϕ*_s_ = 6.11 eV), electrons migrate from the Fe side to the Fe_2_O_3_ side (toward the lower energy state) upon contact, until their Fermi levels reach equilibrium at a common level. Consequently, the metallic Fe region acquires a net positive charge due to electron depletion, while the Fe_2_O_3_ region acquires a net negative charge due to electron accumulation.^[^ ^40^ ^]^ This establishes an electric field at the Fe‐Fe_2_O_3_ interface (Interfacial Electric Field), directed from the metallic Fe side toward the Fe_2_O_3_ oxide side, as illustrated in Figure 6e. Considering the confinement effect of the interfacial electric field on eddy currents (Figure 6e), any electron flow component perpendicular to the interface within an eddy current path would encounter hindrance from the interfacial electric field. Additionally, the Schottky barrier at the metal‐semiconductor interface further impedes electron transport from the metal across the interface.^[^ ^41^ ^]^ As a result, the flow of electrons perpendicular to the interface is significantly reduced; therefore, electrons are more likely to flow parallel to the interface within the metallic Fe region. For the structural units of the Fe‐based nanochain (namely, the Fe‐based nanoparticles), eddy currents become confined within the interior of these nanoparticles, forming closed‐loop paths as illustrated in Figure 6f. In this work, although the naturally formed oxide layers on our as‐prepared Fe/FeCo nanochains contain multiple oxide components (such as Fe_2_O_3_ and Fe_3_O_4_) whose formation cannot be precisely controlled, the work functions of these varied oxides are all significantly higher than that of metallic Fe (including both amorphous Fe oxides and metallic Fe), and they exhibit high resistivity.^[^ ^42^ ^]^ Consequently, the effectiveness of such metal‐oxide interfaces in restricting eddy currents within nanoparticles does not vary significantly due to differences in their composition or structure. This confinement effect endows the Fe‐based nanochain with excellent electrically insulating behavior. Consequently, under high‐frequency conditions, eddy current losses are substantially reduced. Besides, we implemented a homogenization model and employed the finite element method (FEM) to simulate and compare the eddy currents in the Fe nanochain‐SMC and the commercial FeSiBCuNb‐SMC under identical conditions. A detailed account of the parameters and results from the finite element simulations is available in the (Figure S11, Supporting Information).

## 3 Conclusion

In summary, we have successfully addressed the enduring challenge of balancing magnetic coupling and electrical insulation in SMMs through an innovative nanochain engineering strategy. By integrating magnetic field‐directed assembly with in situ oxidation, self‐isolated Fe/FeCo nanochains featuring continuous ultrathin oxide encapsulation (≈10 nm) were obtained. This architecture demonstrates unique advantages for developing high‐frequency soft magnetic devices. First, the oxide layer ensures robust electrical isolation between nanoparticles, elevating resistivity to 0.86 Ω·m (Fe) and 0.41 Ω·m (FeCo), while chain alignment preserves strong exchange coupling, enabling remarkably high *M*_s_ of 149.2 and 172.1 emu g^−1^, respectively, and this magnetic device made of nanochains exhibits remarkable magnetic permeability stability (cut‐off frequency exceeding 1 GHz). Second, theoretical calculations reveal that charge recombination at amorphous‐crystalline interfaces significantly enhances spin polarization, boosting magnetization by 7.9–9.5% compared to homogeneous phases. Thirdly, investigations into the metal‐oxide interface show that the eddy currents are predominantly confined within individual nanoparticles due to interparticle insulation, yielding ultralow core losses. Crucially, these nanochains were chemically synthesized under a low‐intensity magnetic field (10 mT), offering a broad compositional tunability and the potential for large‐scale production. They can be directly compacted into functional inductor cores at low temperature and pressure (100 MPa), without the need for post‐annealing or coating, which represents a significant manufacturing advantage. Furthermore, thermal stability analysis confirms that the Fe/FeCo nanochains maintain considerable stability within the 25–100 °C range, laying a foundation for their application in high‐frequency soft magnetic devices. This work provides significant value to address the inherent trade‐off between magnetic coupling and electrical insulation in high‐frequency soft magnetic materials.

## 4 Experimental Section

### Chemicals

The chemicals utilized were of analytical grade and did not require further purification. Anhydrous ethanol was purchased from Beijing Innochem Co., Ltd. Iron sulfate heptahydrate (FeSO_4_·7H_2_O), cobalt chloride hexahydrate (CoCl_2_·6H_2_O), sodium borohydride (NaBH_4_), and polyvinylpyrrolidone (PVP) were purchased from Shanghai Aladdin Co., Ltd.

### Synthesis of Nanochains

The Fe nanochains and the FeCo nanochains were synthesized via magnetic field‐assisted chemical reduction in a 10 mT uniform magnetic field ($\vec{H}$) generated by Helmholtz coils. Specifically, 0.2 mmol L^−1^ FeSO_4_·7H_2_O (for Fe chains) or FeSO_4_·7H_2_O / CoCl_2_·6H_2_O (Fe: Co = 7:1 molar ratio for FeCo chains) with 1 wt.% PVP stabilizer was dissolved in deionized water. Under constant stirring, 1 mmol L^−1^ NaBH_4_ solution was added dropwise at a speed of 2 mL min^−1^ to initiate reduction. High reaction rates and an excessive concentration of reducing agent adversely affect the morphological homogeneity, leading to compromised uniformity in the nanostructures (both nanoparticles and nanochains). The resulting black magnetic precipitate was collected, ultrasonically washed several times with anhydrous ethanol, and dried in a hot air oven at 50 °C for 12 h. The application of a magnetic field during the reaction enabled magnetic nanochains, whereas zero‐magnetic field controls yielded randomly aggregated nanoparticles.

### Synthesis of Nanochain‐SMCs

Toroidal SMCs were formed by cold‐pressing nanochain powders at room temperature under a pressure of 100 MPa for 5 min. The toroidal SMCs possess an inner diameter of 8 mm, an outer diameter of 13 mm, and a height of ≈2 mm. Toroidal inductors were subsequently fabricated by wrapping insulated conductive wires around toroids.

### Material Characterization

The phase structures of nanochains were characterized by X‐ray diffraction (XRD, Bruker D8 ADVANCE). The morphology of nanochains was observed by scanning electron microscopy (SEM, Zeiss Sigma 300). The microstructures of the nanochain‐SMCs were observed by transmission electron microscopy (TEM, Talos F200X G2) following focused ion beam milling (Helios 6 HD FIB‐SEM). The elemental distribution was characterized by a high‐angle annular dark‐field detector (HAADF). The surface chemical states of nanochains were characterized by X‐ray photoelectron spectroscopy (XPS, Thermo Scientific K‐Alpha). The thermal stability of the Fe/FeCo nanochains was investigated by simultaneous TG‐DSC (HITACHI STA200) analysis. Magnetic hysteresis loops and the Magnetization‐Temperature curve were measured by the magnetic property measurement system (SQUID, MPMS, Quantum Design 3). The resistivity of nanochain‐SMCs was measured by the four‐wire method (PPMS Dynacool 9T). The permeability and quality factor were measured by an impedance analyzer (E4991A, Agilent). The core loss and dynamic coercivity of nanochain‐SMCs were measured with a B‐H analyzer (SY‐8219).

### First‐Principle Calculation

The calculations were conducted within the framework of spin‐polarized density functional theory (DFT) as implemented in the Vienna Ab‐initio Simulation Package (VASP 6.3.0). The Perdew‐Burke‐Ernzerhof (PBE) version of generalized‐gradient approximation (GGA) was adopted to describe the exchange‐correlation interaction among electrons. Hubbard U correction was included with effective U values of 5.0 eV for Fe‐3d orbitals. The plane wave cut‐off energy was set to 420 eV. The k‐point mesh was generated using the Monkhorst‐Pack scheme with a grid spacing of 0.04 Å^−1^. For amorphous, crystalline, and composite structures, the lattice parameters and atomic positions were fully relaxed until the variation of total energy was within 10^−5^ eV and the final force on each atom was less than 0.01 eV Å^−1^. The amorphous model was obtained via melt‐rapid quenching of the corresponding crystal model by molecular dynamics (MD) simulation. Periodic boundary conditions were applied, with 8 Å vacuum layers introduced on both sides of the composite structural model. All MD calculations were conducted within a canonical NVT ensemble, which entails a constant number of particles, volume, and temperature. The temperature regulation was achieved through the Nose‐Hoover thermostat.

### FEM Simulation

3D toroidal inductor simulations incorporated experimentally measured material parameters (B‐H curve, core loss, density, resistivity, and permeability). The FEM simulation process was realized through ANSYS Maxwell.

## Conflict of Interest

The authors declare no conflict of interest.

## Author Contributions

D. Z. and J. Z. conceived the idea and designed the experiment. D. Z. and R. B. carried out the experiments. D. Z. and L. L. performed the calculation and simulation. D. Z. and H. D. conducted the material characterization. D. Z. wrote the original draft. H. K. and P. Y. revised the manuscript. H. K. and W. W. supervised the project. All authors discussed the results and contributed to the manuscript.

## Supporting information



Supporting Information

## Data Availability

The data that support the findings of this study are available from the corresponding author upon reasonable request.
