# Supplementary material for: Overcoming the Trade‐Off Between Magnetic Coupling and Electrical Insulation in Soft Magnetic Materials via Nanochain Engineering
Source: Adv Sci (Weinh). 2025 Dec 16:e17270. Online ahead of print. doi: 10.1002/advs.202517270 (PMC13325503; doi:10.1002/advs.202517270)
Supplement: Supplementary file 1 — Supporting Information [file ADVS-9999-e17270-s001.docx]

Supporting Information

**Overcoming the Trade-off Between Magnetic Coupling and Electrical Insulation in Soft Magnetic Materials via Nanochain Engineering**

*Dingrong Zuo, Lichen Liu, Haibo Ke,* Rongsheng Bai, Huaping Ding, Jing Zhou,* Zhenxiang Cheng,* Peng Yu,* and Weihua Wang*

D. Zuo, H. Ke, P. Yu

College of Physics and Electronic Engineering, Chongqing Normal University, Chongqing 401331, China

E-mail: [pengyu@cqnu.edu.cn](mailto:pengyu@cqnu.edu.cn) (P. Yu)

D. Zuo, L. Liu, H. Ke, R. Bai, H. Ding, J. Zhou, W. Wang

Songshan Lake Materials Laboratory, Dongguan 523808, China

E-mail: [kehaibo@sslab.org.cn](mailto:kehaibo@sslab.org.cn) (H. Ke)

E-mail: [zhoujing@sslab.org.cn](mailto:zhoujing@sslab.org.cn) (J. Zhou)

Z. Cheng

Institute for Superconducting and Electronic Materials, Faculty of Engineering and Information Sciences, University of Wollongong, Innovation Campus, Squires Way, North Wollongong, NSW 2500, Australia

E-mail: [cheng@uow.edu.au](mailto:cheng@uow.edu.au) (Z. Cheng)

W. Wang

Institute of Physics, Chinese Academy of Sciences, Beijing 100190, China

Keywords: Nanochain engineering, Nanoparticles, Magnetic coupling, Electrical insulation, High-frequency soft magnetic materials, Soft magnetic composites

**CONTENTS:**

1. **Elemental mapping of the Fe/FeCo nanochains**
2. **TEM and HAADF analysis of the FeCo nanochains**
3. **Co 2p XPS spectra of the FeCo nanochains**
4. **SEM images of the Fe/FeCo nanoparticles**
5. **TG-DSC thermal analysis of the Fe nanoparticles and the Fe nanochains**
6. **M-T curve of the Fe/FeCo nanochains**
7. **Coercivity analysis of the Fe/FeCo nanochain-SMCs**
8. **Analysis of the core loss composition for the Fe/FeCo nanochain-SMCs**
9. **Table of the *M*_s_ and *ρ* data for the soft magnetic composite materials**
10. **Amorphous metal-oxide (Fe-Fe_2_O_3_) interface model**
11. **PDOS of the amorphous Fe, Fe_2_O_3_, and Fe-Fe_2_O_3_**
12. **FEM simulation of the Fe nanochain-SMC**
13. **Elemental mapping of the Fe nanochains and the FeCo nanochains**


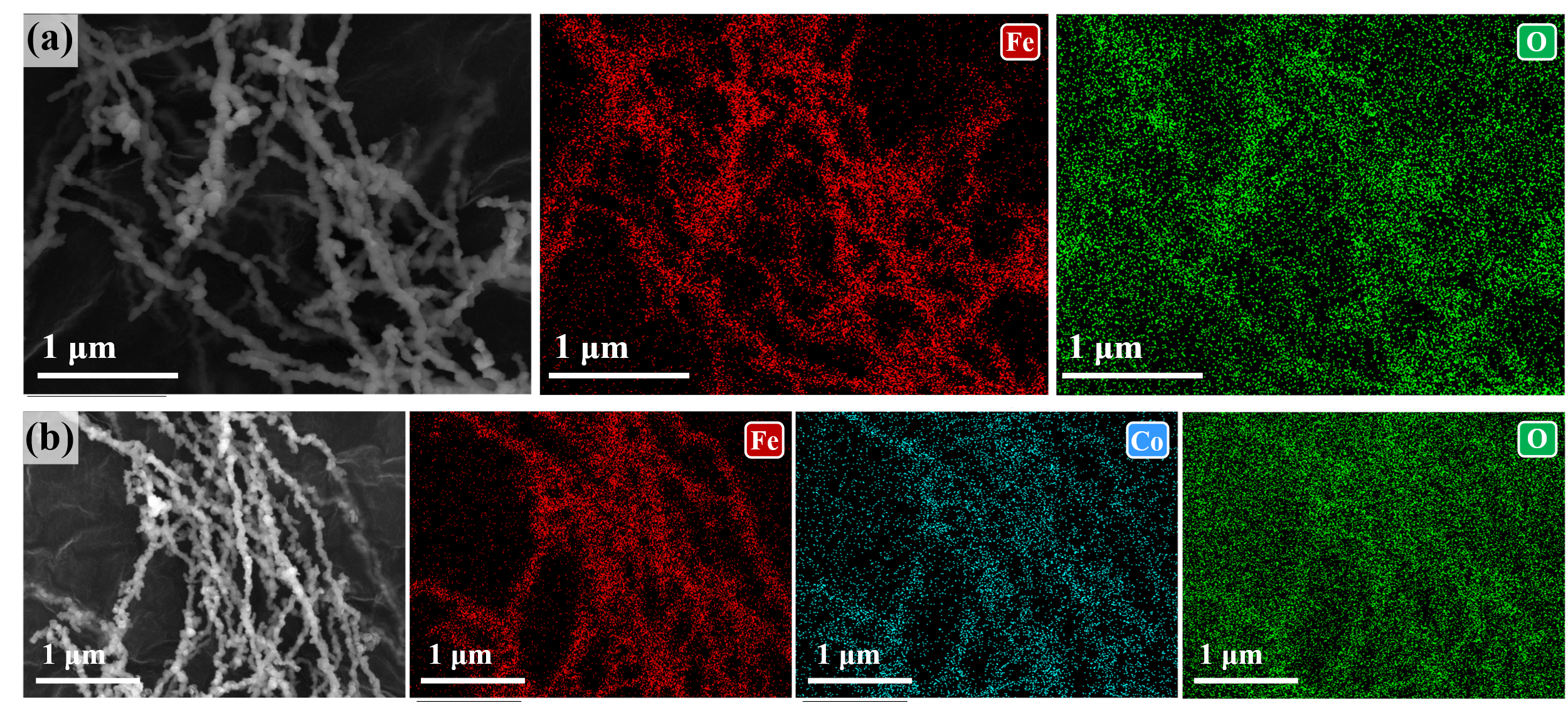
Figure S1a shows the elemental distribution of Fe nanochains, and Figure S1b shows the FeCo nanochains.

**Figure S1.** a) Elemental mapping of the Fe nanochains. b) Elemental mapping of the FeCo nanochains.

1. **TEM and HAADF analysis of the FeCo nanochains**

TEM and HAADF examination of FIB-prepared samples provides detailed microstructural insights into both FeCo nanochains and FeCo nanochain-SMCs. Figure S2a shows the microscopic morphology of the FeCo nanochains. Figure S2b shows the FFT (numbers 1, 3, and 4) and HR-TEM (number 2) images of the different regions marked as the blue square in Figure S2a. Figure S2c shows the HAADF images and elemental maps (scalebar represents 200 nm) of the FeCo nanochains-SMC. Figure S2d shows the elemental composition of the FeCo nanoparticle crossed by the blue arrow in Figure S2c.


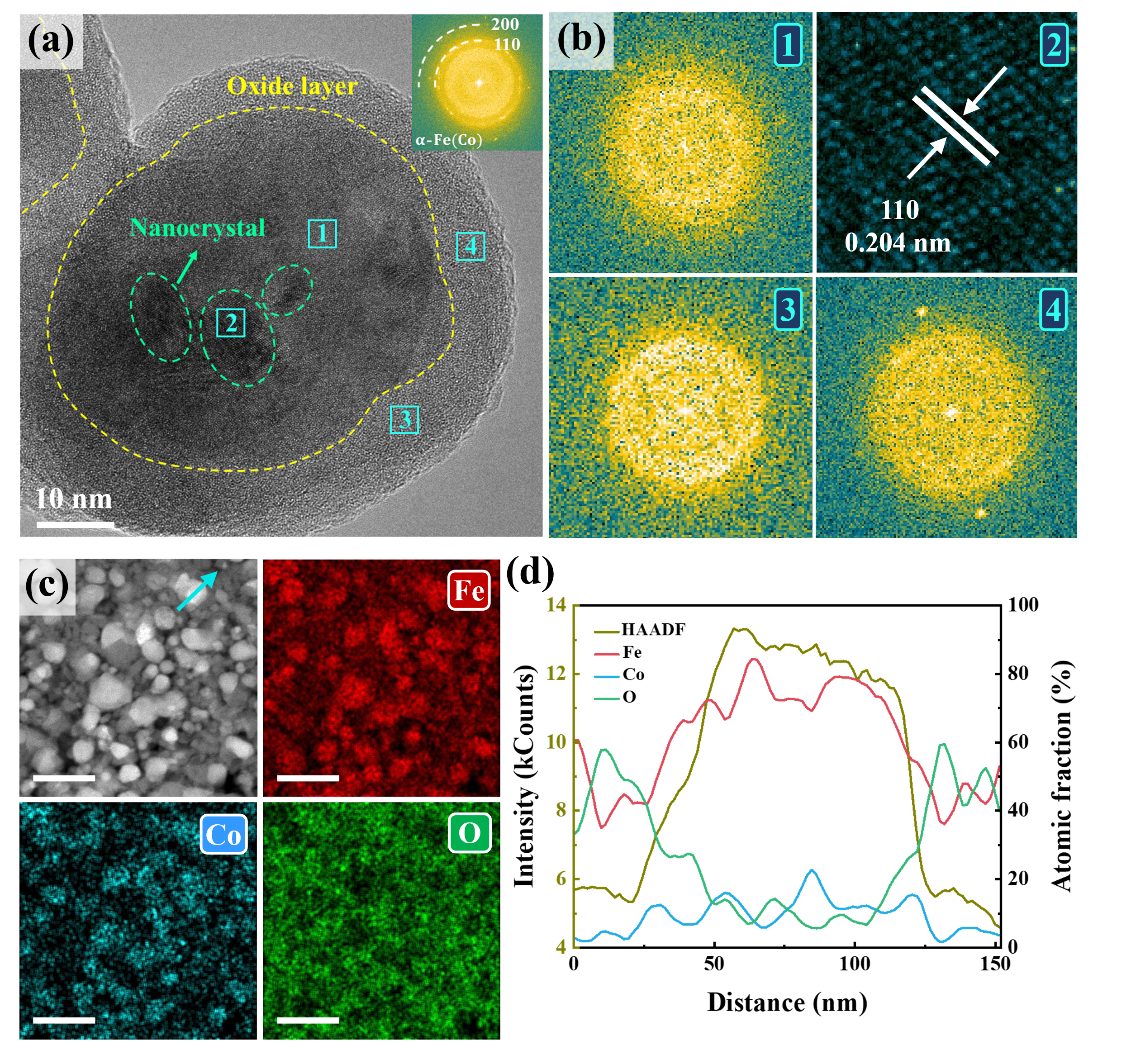


**Figure S2.** a) TEM images of the FeCo nanochains. b) FFT (numbers 1, 3, and 4) and HR-TEM (number 2) images for the different regions marked as the blue square in (a). c) HAADF images and elemental maps (scalebar represents 200 nm) of the FeCo nanochains-SMC. d) Elemental composition of the FeCo nanoparticle crossed by the blue arrow in (c).

1. **Co 2p XPS spectra of the FeCo nanochains**

Figure S3 indicates that the Co element in the FeCo nanochains exists in both 0, +2, and +3 valence states.


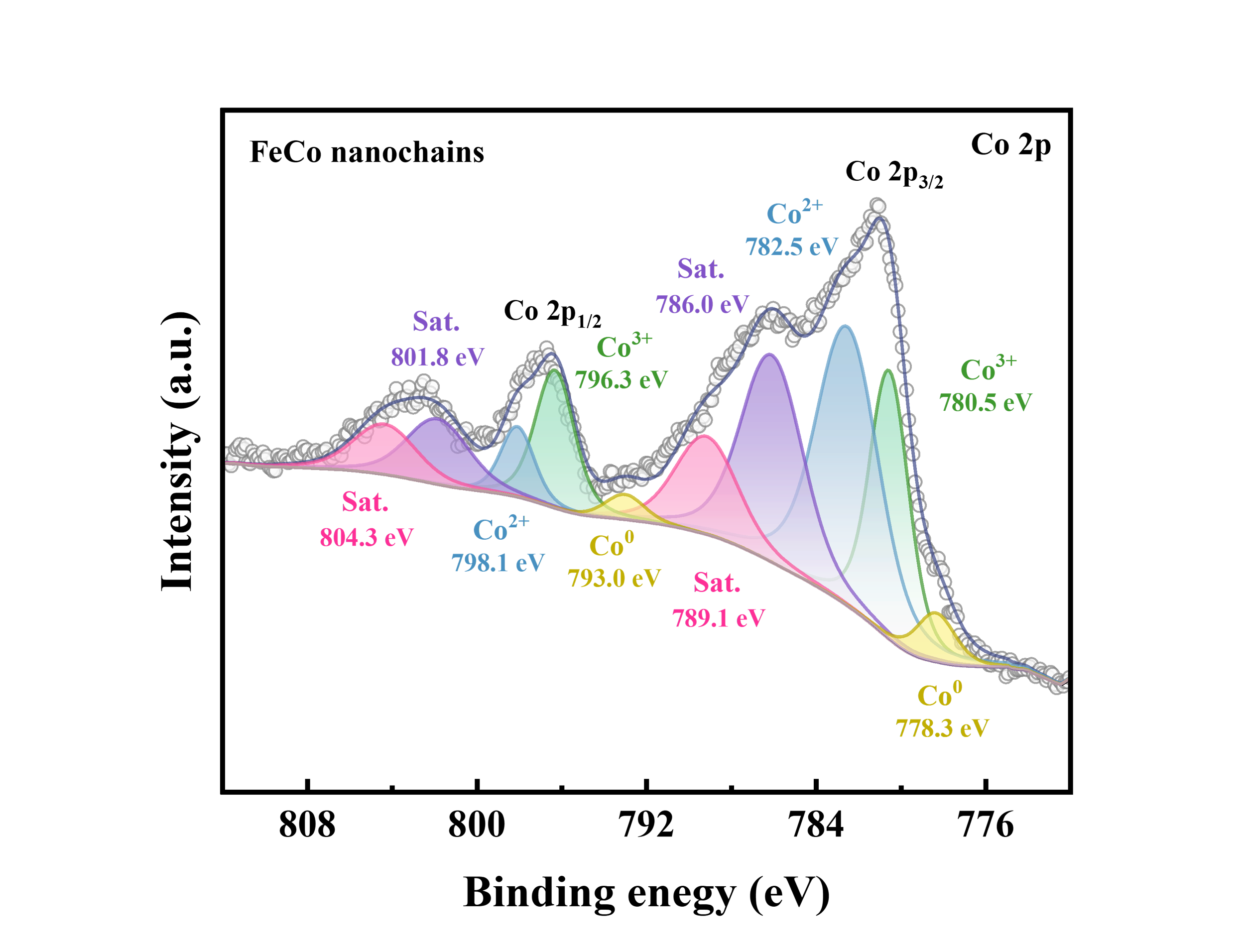


**Figure S3.** Co 2p XPS spectra of the FeCo nanochains.

1. **SEM images of the Fe/FeCo nanoparticles**

Figure S4 presents the disordered clusters of Fe (Figure S4a) and FeCo (Figure S4b) nanoparticles, synthesized in the absence of an applied magnetic field during reduction.


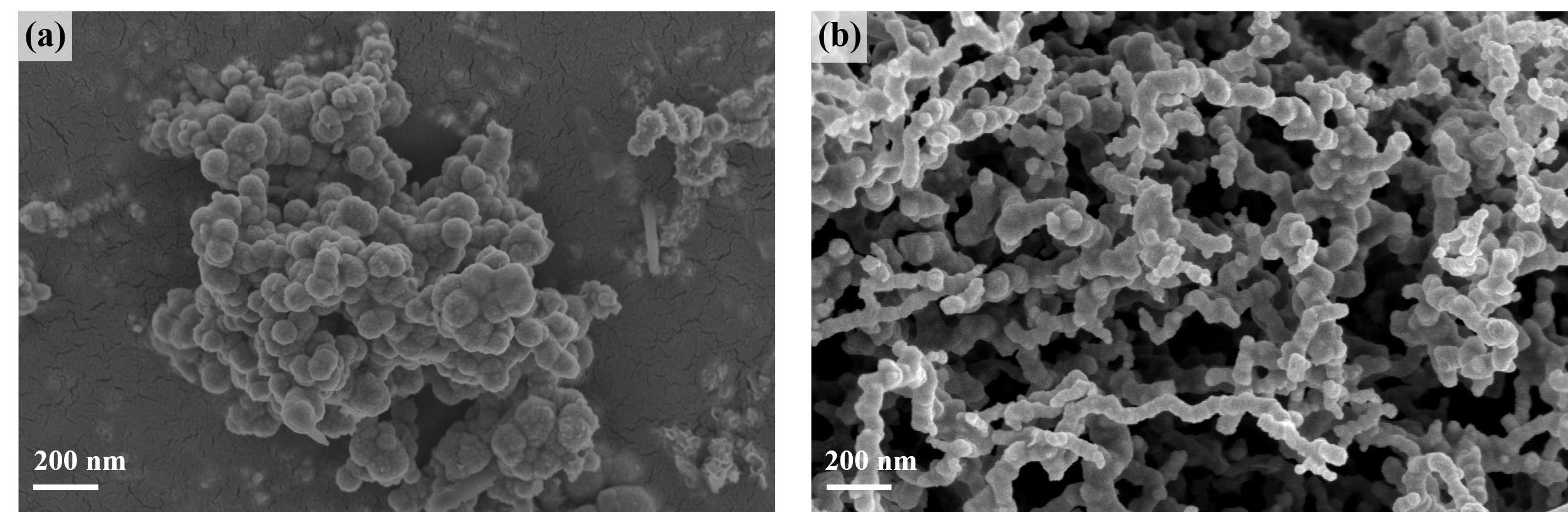


**Figure S4.** SEM images demonstrating the nanoparticle with disordered clusters. (a) Fe nanoparticles. (b) FeCo nanoparticles.

1. **TG-DSC thermal analysis of the Fe nanoparticles and the Fe nanochains**

Figure S5 presents the TG, DTG, and DSC curves of Fe nanoparticles (Figure S5a) and Fe nanochains (Figure S5b), respectively, measured during heating from 300 K to 800 K under an Ar atmosphere at a heating rate of 10 K/min.


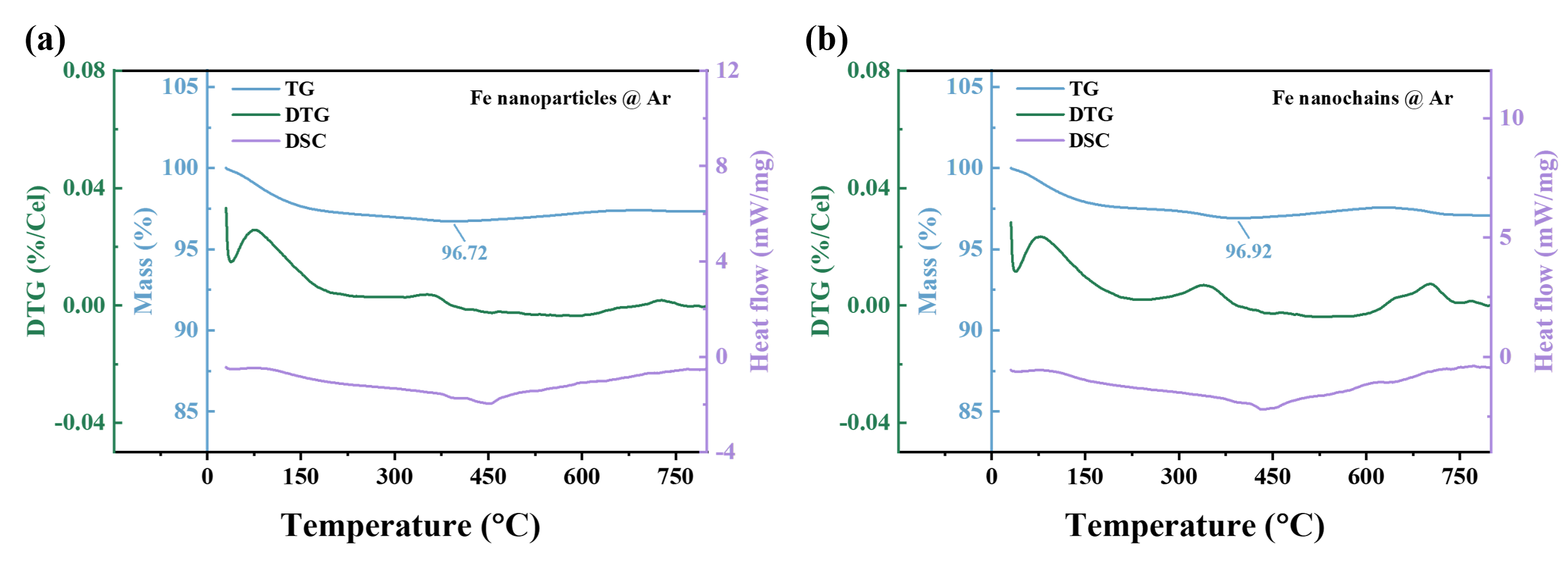


**Figure S5.** Simultaneous TG-DSC thermal analysis of the Fe nanoparticles (a) and the Fe nanochains (b).

1. **M-T curve of the Fe/FeCo nanochains**

Figure S6 presents the magnetization-temperature (M-T) curve of the Fe/FeCo nanochains.


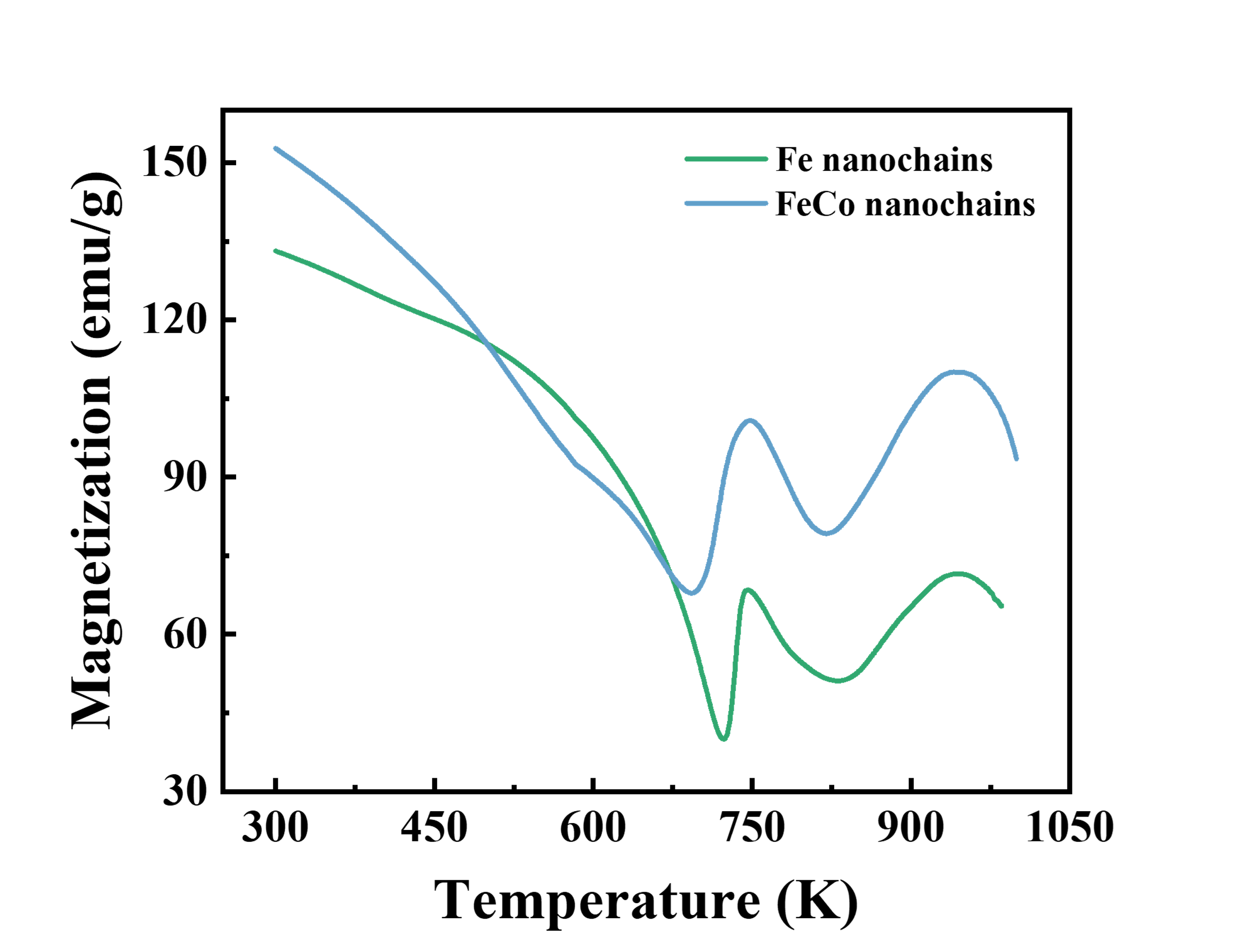


**Figure S6.** Magnetization-Temperature (M-T) curve of the Fe/FeCo nanochains.

1. **Coercivity analysis of the Fe/FeCo nanochain-SMCs**

Figure S7 displays the coercivity of the Fe/FeCo nanochain-SMCs at different frequencies. The Fe/FeCo nanochain-SMCs maintain considerably low coercivity within the 300-1000 kHz range, and the coercivity gradually plateaus with increasing frequency.


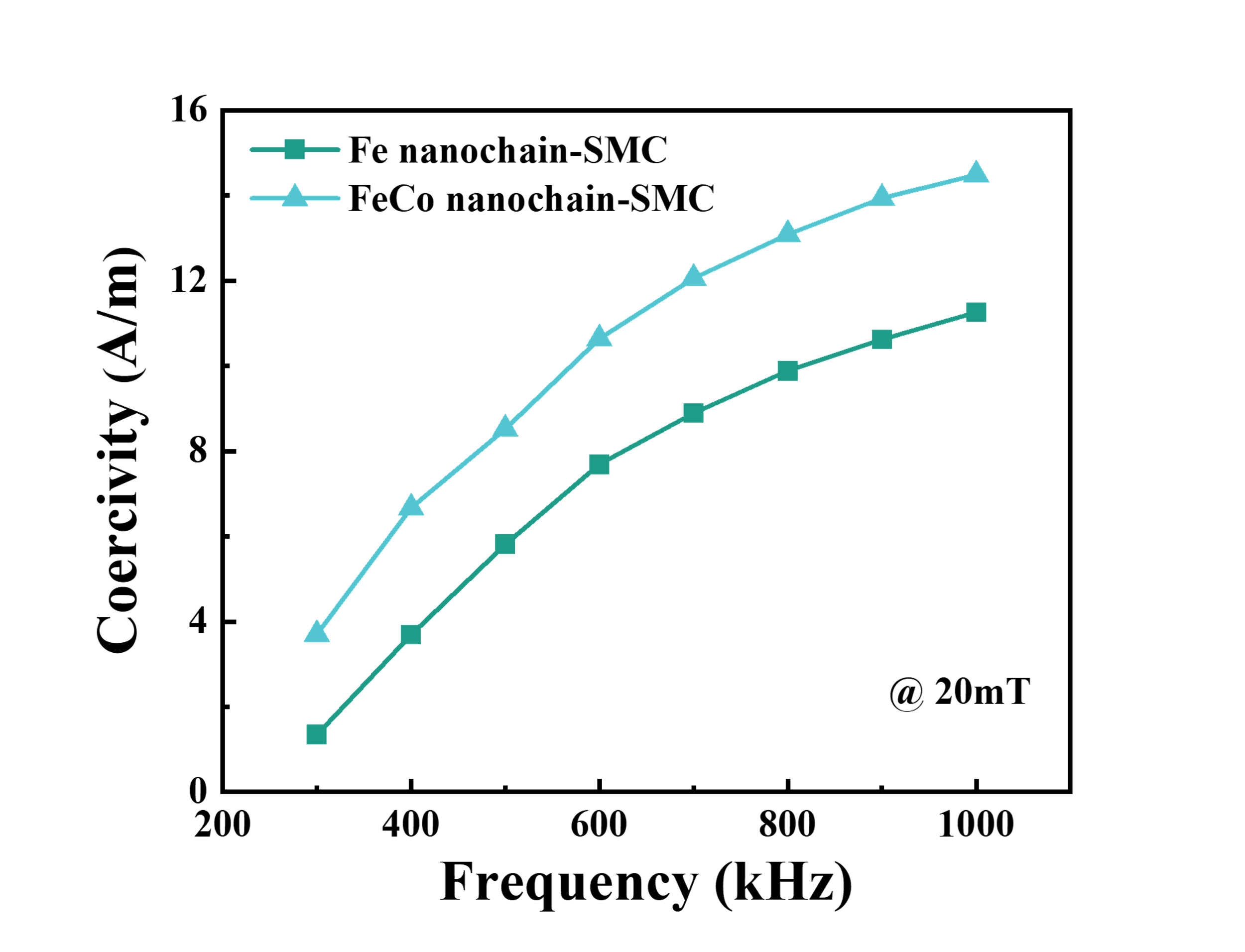


**Figure S7.** The Fe/FeCo nanochain-SMCs at different frequencies.

1. **Analysis of the core loss composition for the Fe/FeCo nanochain-SMCs**


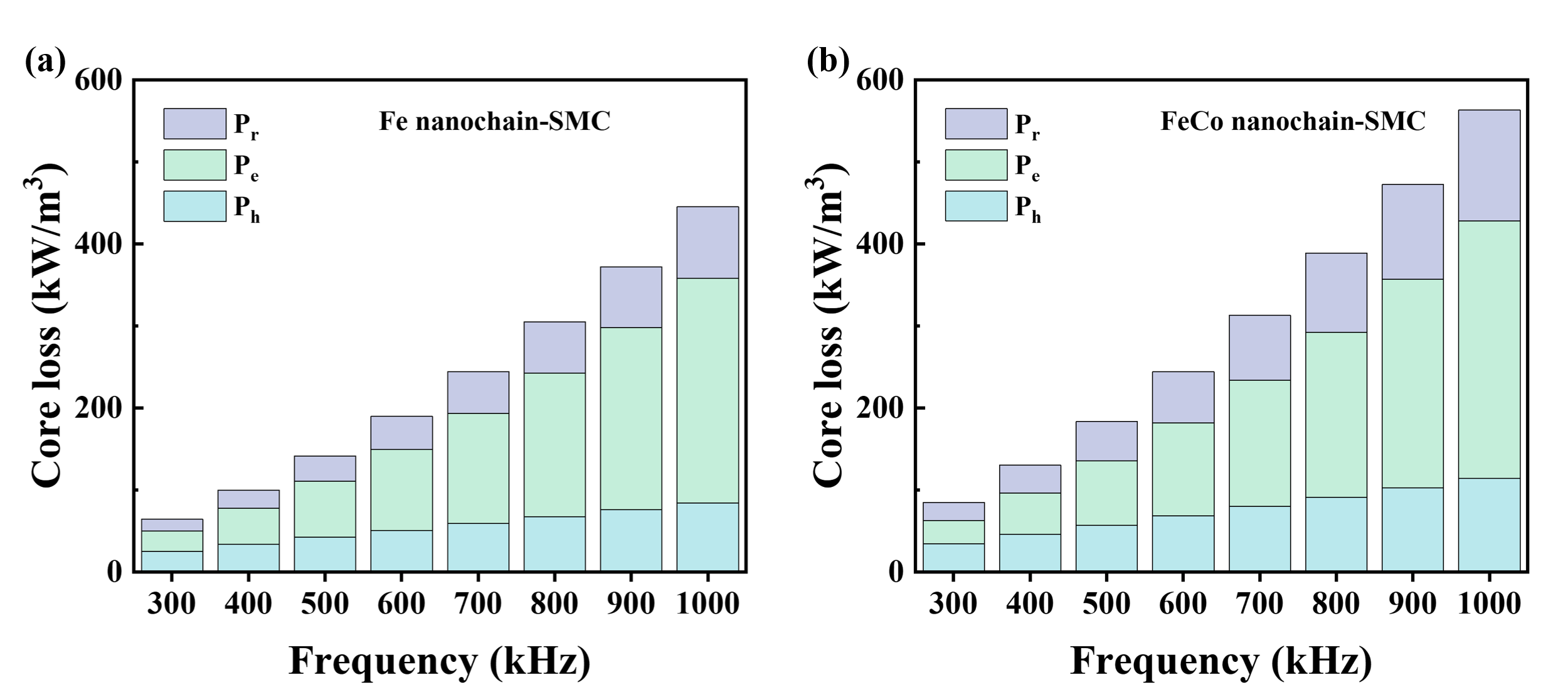
Figure S8 shows the core loss composition of the Fe nanochain-SMC (Figure S8a) and the FeCo nanochain-SMC (Figure S8b) in the frequency range of 300 to 1000 kHz.

**Figure S8.** The core loss composition of the Fe nanochain-SMC (a) and the FeCo nanochain-SMC (b) at different frequencies, the *B*_m_ = 20 mT.

According to the conventional Steinmetz model, the total power loss *P*_tot_ in typical soft magnetic materials can be approximately divided into three components:

$$\begin{aligned} P_{tot}=P_{h}+P_{e}+P_{r}=K_{h}\cdot f+K_{e}\cdot f^{2}+K_{r}\cdot f^{1.5}\#S\left( 1 \right) \end{aligned}$$

where *P*_tot_ is the total core loss, Ph is the hysteresis loss, *P*_e_ is the eddy current loss, *P*_r_ is the residual loss, $f$ is the frequency, *K*_h_ is the hysteresis loss coefficient, *K*_e_ is the eddy current loss coefficient, and *K*_r_ is the residual loss coefficient. Generally, the non-negative loss coefficients (*K*_h_, *K*_e_, *K*_r_) were obtained by fitting the model to the experimentally measured core loss data using the non-negative least squares (NNLS) method. Then, based on the aforementioned loss coefficients, the loss components of the material at different frequencies can be calculated, the results as shown in Figure S8a (Fe nanochain-SMC) and Figure S8b (FeCo nanochain-SMC). Table S1 and Table S2 list the proportion of various losses for the Fe and FeCo nanochain-SMC, respectively, within the frequency range of 300-1000 kHz. The results demonstrate that as the frequency increases from 300 kHz to 1000 kHz, the proportion of hysteresis loss and residual loss in Fe/FeCo nanochain-SMCs gradually decreases, while the proportion of eddy current loss progressively increases and becomes the predominant contributor (Fe: 61.55%; FeCo: 55.77%).

**Table S1. Proportion of various losses of the Fe nanochain-SMC.**

| **Frequency (kHz)** | ***P*_tot_ (kW/m^3^)** | ***P*_h_ (%)** | ***P*_e_ (%)** | ***P*_r_ (%)** |
| --- | --- | --- | --- | --- |
| 300 | 63.28 | 39.34 | 38.41 | 22.25 |
| 400 | 98.18 | 33.84 | 44.05 | 22.11 |
| 500 | 139.79 | 29.79 | 48.46 | 21.75 |
| 600 | 190.93 | 26.65 | 52.03 | 21.32 |
| 700 | 241.26 | 24.14 | 54.99 | 20.87 |
| 800 | 299.08 | 22.09 | 57.51 | 20.40 |
| 900 | 369.36 | 20.37 | 59.67 | 19.96 |
| 1000 | 446.86 | 18.91 | 61.55 | 19.54 |

**Table S2. Proportion of various losses of the FeCo nanochain-SMC.**

| **Frequency (kHz)** | ***P*_tot_ (kW/m^3^)** | ***P*_h_ (%)** | ***P*_e_ (%)** | ***P*_r_ (%)** |
| --- | --- | --- | --- | --- |
| 300 | 84.23 | 40.40 | 33.39 | 26.21 |
| 400 | 129.68 | 35.08 | 38.65 | 26.27 |
| 500 | 181.49 | 31.11 | 42.84 | 26.05 |
| 600 | 246.75 | 28.01 | 46.29 | 25.7 |
| 700 | 311.86 | 25.52 | 49.20 | 25.28 |
| 800 | 389.13 | 23.46 | 51.69 | 24.85 |
| 900 | 472.81 | 21.73 | 53.86 | 24.41 |
| 1000 | 564.43 | 20.25 | 55.77 | 23.98 |

1. **Table of the *M*_s_ and *ρ* data for the soft magnetic composite materials**

**Table S3. *M*_s_ and *ρ* data for the soft magnetic composite materials**

| **Samples** | ***M*_s_ (emu/g)**  **or *B*_s_ (T)** | ***ρ* (μΩ·cm)** | **Ref.** |
| --- | --- | --- | --- |
| Fe@Fe3O4/ZrO2 | 1.2 T | 1.1 × 10^4^ | [1] |
| Fe@SiO2 | 154 emu/g | 1.4 × 10^3^ | [2] |
| Fe@Resin | 0.64 T | 1.6 × 10^5^ | [3] |
| Fe@PA | 1.4 T | 1.07 × 10^4^ | [4] |
| FeSi@Mn-SiO2 | 186 emu/g | 5.2 × 10^3^ | [5] |
| FeSi@Fe2SiO4 | 188 emu/g | 3.4 × 10^3^ | [6] |
| FeSi@Bi2O3 | 1.9 T | 8 × 10^4^ | [7] |
| FeSiAl@SiO2 | 125 emu/g | 9.7 × 10^5^ | [8] |
| FeSiAl@AIN/Al2O3 | 143 emu/g | 1.1 × 10^4^ | [9] |
| FeSiCr@Ni-Zn Ferrite | 157 emu/g | 1.25 × 10^4^ | [10] |
| FeSiBP@Ni-Zn Ferrite | 156 emu/g | 1.5 × 10^3^ | [11] |

1. **Amorphous metal-oxide (Fe-Fe_2_O_3_) interface model**

Figure S9 displays the amorphous Fe-Fe₂O₃ interface model constructed by molecular dynamics (MD) simulation.


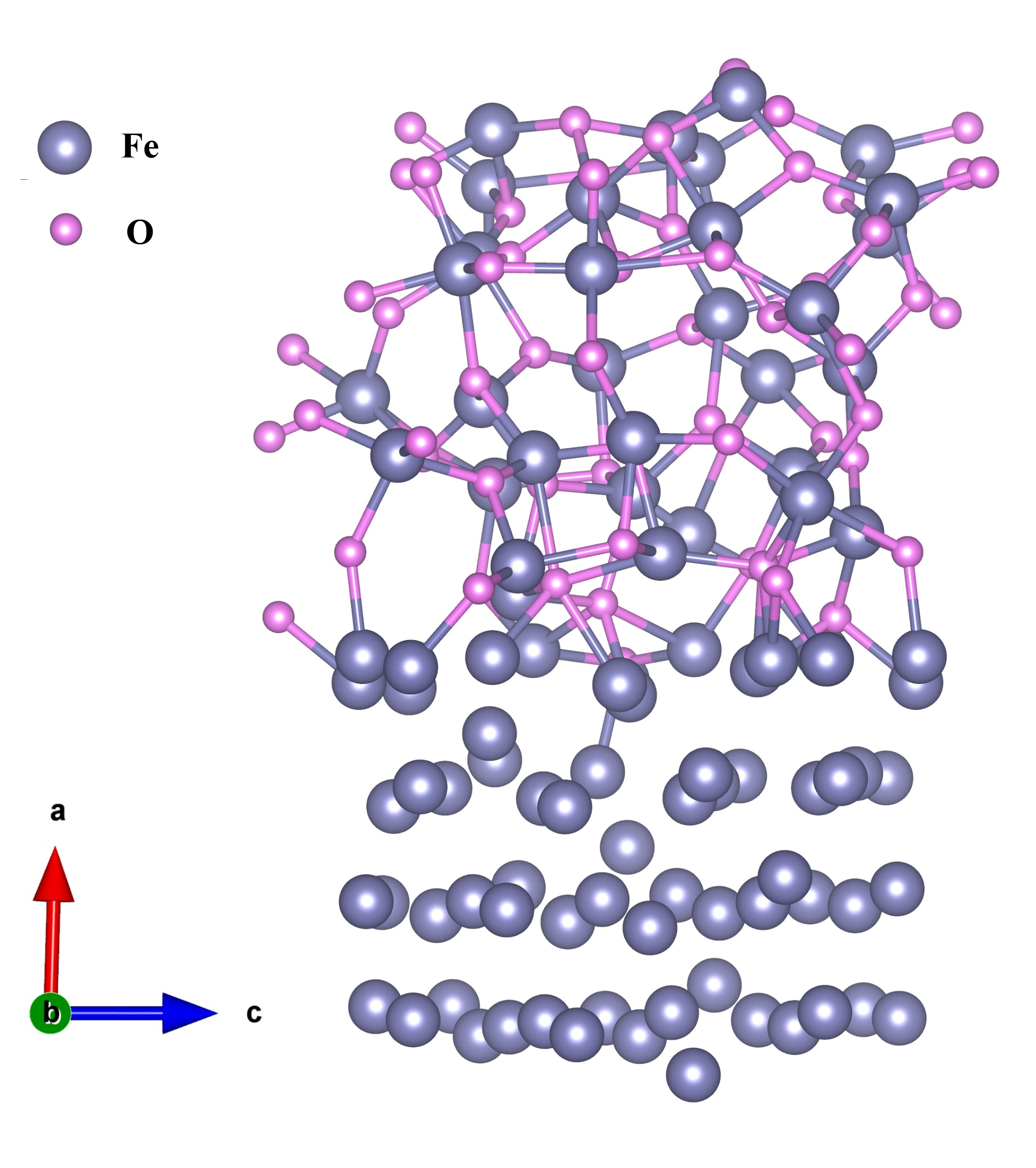


**Figure S9.** Amorphous metal-oxide (Fe-Fe_2_O_3_) interface model.

1. **PDOS of the amorphous Fe, Fe_2_O_3_, and Fe-Fe_2_O_3_**

Figure S10 shows the partial density of states (PDOS) of each component in the model from Figure S9: the amorphous Fe phase, the amorphous Fe₂O₃ phase, and the complete Fe-Fe₂O₃ interface structure.

**
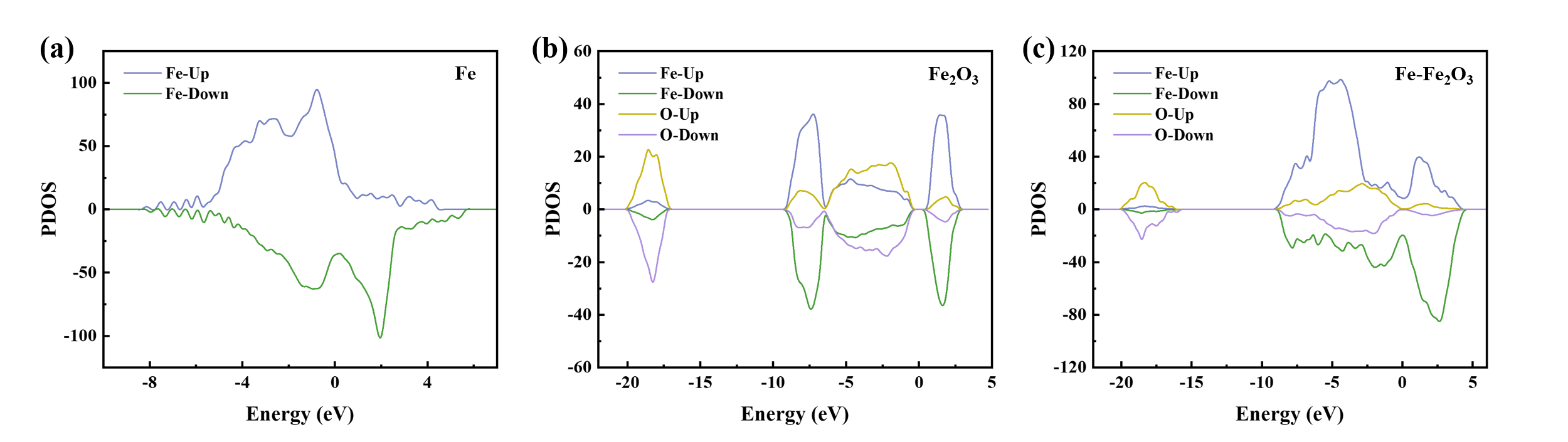
**

**Figure S10.** Partial Density of States (PDOS) of the amorphous Fe (a), Fe_2_O_3_ (b), and Fe-Fe_2_O_3_ (c) structure.

1. **FEM simulation of the Fe nanochain-SMC**

The model integrates experimentally determined parameters – including permeability, resistivity, relative permittivity, B-H curve, core loss characteristics, and density of materials, while also considering the skin effect. As validated in Figure S11a, a 3D toroidal inductor (inner diameter: 8 mm; outer diameter: 13 mm; Thickness: 2 mm) employing the Fe nanochain-SMC parameters demonstrates substantially suppressed eddy currents versus commercial FeSiBCuNb-SMC at 10 MHz excitation (*B*_m_ = 20 mT). Quantitative comparison of eddy current distributions reveals vortex magnitude in the Fe nanochain-SMCs (Figure S11c) is approximately 1/8 that of conventional SMCs (Figure S11b), confirming superior high-frequency applicability.


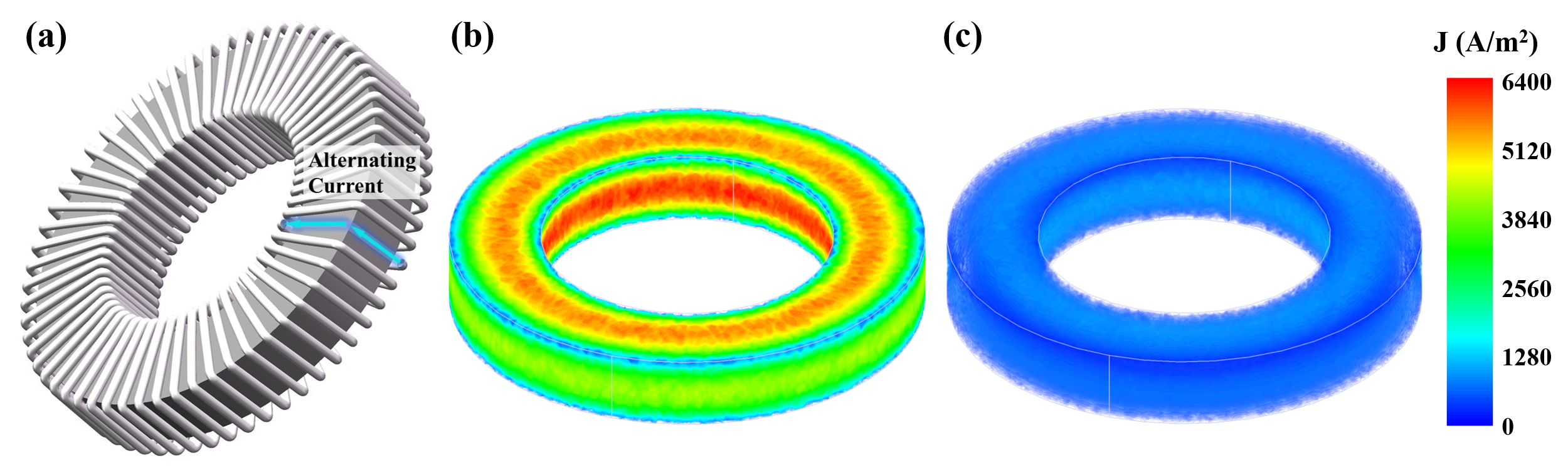


**Figure S11.** Finite Element Method (FEM) simulation of the ring sample. (a) Three-dimensional modeling of toroidal inductors (60 turns; inner diameter: 8 mm; outer diameter: 13 mm; Thickness: 2 mm). (b) and (c) Distribution of eddy current density in toroidal inductors fabricated by the FeSiBCuNb SMC (b) and the Fe nanochain-SMC (c) at 10 MHz, and *B*_m_ = 20 mT.

[1] W. Li, Z. Wang, Y. Ying, J. Yu, J. Zheng, L. Qiao, S. Che, *Ceram. Int.* **2019**, *45*, 3864.

[2] Z. Wu, X. Fan, J. Wang, G. Li, Z. Gan, Z. Zhang, *J. Alloys Compd.* **2014**, *617*, 21.

[3] M. M. Dias, H. J. Mozetic, J. S. Barboza, R. M. Martins, L. Pelegrini, L. Schaeffer, *Powder Technol.* **2013**, *237*, 213.

[4] G. Zhang, G. Shi, W. Yuan, Y. Liu, *Ceram. Int.* **2021**, *47*, 8795.

[5] Z. Luo, X. Fan, Y. Zhang, Z. Yang, J. Wang, Z. Wu, X. Liu, G. Li, Y. Li, *Adv. Powder Technol.* **2021**, *32*, 3364.

[6] Z. Luo, X. Fan, Y. Zhang, Z. Yang, J. Wang, Z. Wu, X. Liu, G. Li, Y. Li, *J. Alloys Compd.* **2021**, *862*, 158595.

[7] P. W. Huang, J. Liu, H. L. Fang, *Mater. Des.* **2022**, *219*, 110755.

[8] W. Li, H. Cai, Y. Kang, Y. Ying, J. Yu, J. Zheng, L. Qiao, Y. Jiang, S. Che, *Acta Mater.* **2019**, *167*, 267.

[9] B. Zong, Y. Wu, P. Ho, W. Chan, Y. Yang, C. Zhao, T. Deng, N. Phuoc, Z. Li, *J. Alloys Compd.* **2018**, *730*, 284

[10] R. Guo, S. Wang, Z. Yu, K. Sun, X. Jiang, G. Wu, C. Wu, Z. Lan, *J. Alloys Compd.* **2020**, *830*, 154736.

[11] S. Lu, M. Wang, Z. Zhao, *J. Non-Cryst. Solids* **2023**, *616*, 122440.
